# Real-Time Predictors of Return of Spontaneous Circulation in an Emergency Setting: A Five-Year Retrospective Study

**DOI:** 10.3390/diagnostics15172202

**Published:** 2025-08-29

**Authors:** Burcu Bayramoglu, Ismail Kaftanci, Ismail Tayfur, Ramazan Guven, Sinem Guzel Ozturk, Betul Kaplan Zamanov, Berna Atli Dasdelen

**Affiliations:** 1 Department of Emergency Medicine, Sancaktepe Sehit Prof. Dr. Ilhan Varank Research and Training Hospital, University of Health Sciences, 34785 Istanbul, Türkiye; 2 Department of Emergency Medicine, Istanbul Cam and Sakura City Research and Training Hospital, University of Health Sciences, 34480 Istanbul, Türkiye; 3 Department of Emergency Medicine, Yenişehir State Hospital, 16900 Bursa, Türkiye; 4 Department of Emergency Medicine, Göztepe Süleyman Yalçın City Hospital, 34722 Istanbul, Türkiye

**Keywords:** cardiac arrest, cardiopulmonary resuscitation, resuscitation, return of spontaneous circulation

## Abstract

**Background**: Cardiopulmonary resuscitation (CPR) is a highly effort-intensive intervention and, in cases of cardiac arrest, the ability to predict a return of spontaneous circulation (ROSC) is of great importance for the efficient use of resources. This real-time assessment approach offers a practical advantage by increasing the applicability of prognostic models during acute resuscitation in an emergency department. **Method**: In this study, the data of patients who underwent CPR in the emergency department of a tertiary care hospital between 1 June 2019 and 1 June 2024 and underwent cardiopulmonary resuscitation were retrospectively analyzed. The patients’ demographics, comorbidities, CPR characteristics, and laboratory findings were evaluated using logistic regression and ROC curve analysis to identify the predictors of ROSC. **Result**: Our study revealed that cases with shockable rhythms and a shorter CPR duration were more likely to achieve ROSC. Elevated levels of albumin, creatine kinase, glucose, hemoglobin, and white blood cells were significantly associated with ROSC, while higher levels of creatinine, base excess, and eosinophils were more common in non-survivors. Atrial fibrillation and neurodegenerative disease were associated with lower ROSC rates. **Conclusions**: Although the criteria for the termination of cardiac arrest resuscitation are not definitive, certain patient characteristics and laboratory findings may guide the prediction of ROSC or the identification of cases requiring prolonged CPR. The integration of these real-time predictors into clinical algorithms may support decision making in crowded emergency departments.

## 1. Introduction

According to the current American Heart Association guidelines, unresponsive patients with abnormal or absent breathing and those without a detectable pulse for over 10 s during a pulse check by healthcare professionals should be considered to be in cardiac arrest, and cardiopulmonary resuscitation (CPR) should be initiated [1]. CPR is an intervention that requires considerable physical effort and, in crowded emergency departments, when multiple patients simultaneously require CPR, assigning a large number of healthcare professionals to a single cardiac arrest case may compromise the care of other patient groups. Therefore, predicting ROSC in cardiac arrest cases is of great significance for the efficient use of resources.

Although prognostic models such as the Pre-Arrest Morbidity, Cardiac Arrest Survival Post-Resuscitation In-Hospital Score, the Risk Stratification Score for Resuscitation, and the Good Outcome Following Attempted Resuscitation 2 have previously been developed for predicting ROSC, most of these models primarily focus on parameters related to pre-arrest clinical data [2]. A significant limitation in regard to the practical application of existing ROSC prediction models is their reliance on patients’ known comorbidities. However, in an emergency department setting, most cardiac arrest cases, particularly involving those presenting with out-of-hospital cardiac arrest, arrive without an accessible medical history, rendering such models less applicable. Distinct from prior research, our study aims to identify ROSC predictors that are immediately assessable during resuscitation, independent of the patient’s preexisting medical records. This real-time approach may enhance the utility of prognostic tools in acute emergency care contexts.

This study aimed to identify variables that may be used to predict ROSC in patients experiencing cardiac arrest. In this way, appropriate interventions can be prioritized for patients with higher ROSC probability, while in cases with low ROSC probability, the CPR duration can be adjusted on a patient-specific basis, the workforce loss can be minimized, and other patients in the emergency department can receive adequate treatment.

## 2. Materials and Methods

### 2.1. Study Design

In this study, the data of patients who presented to the emergency department of a tertiary care hospital between 1 June 2019 and 1 June 2024, and who underwent cardiopulmonary resuscitation were retrospectively analyzed. The hospital where the study was conducted is a training and research hospital, operating across three separate campuses. Cardiac arrest cases brought in by emergency aid services are immediately taken to the resuscitation room, where resuscitation is continued. For cases in which the patient is developing cardiac arrest during emergency department follow-up, chest compressions are initiated, a mechanical compression device is applied, and advanced airway management is secured, before transferring the patient to the resuscitation room.

CPR was performed in accordance with the current American Heart Association guidelines [1]. Chest compressions were administered using mechanical compression devices, with the LUCAS 2 and LUCAS 3 (Jolife AB, Lund, Sweden) brands and models. In cases where mechanical compression devices were not suitable, manual compressions were applied.

### 2.2. Study Population

Patients aged 18 years and above who received CPR were included in the study. Patients under 18 years of age, pregnant women, those with traumatic arrest, and patients with missing data were excluded from the study.

### 2.3. Data Collection

From the hospital information management system, the following data were recorded and statistically analyzed: the patients’ demographic information; their mode of arrival and presenting complaints; chronic diseases; duration of prehospital (CPR duration performed by the emergency response team) and in-hospital CPR if administered; total duration of CPR (in and out of hospital); rhythms observed during CPR; CPR outcome; rhythm observed if ROSC was achieved; complete blood count (white blood cell [WBC], neutrophil, monocyte, lymphocyte, eosinophil, red blood cell [RBC], hemoglobin, hematocrit, and platelet levels); biochemical parameters (albumin, alanine aminotransferase, aspartate aminotransferase, creatine kinase [CK], glucose, calcium, chloride, creatinine, lactate dehydrogenase, potassium, sodium, protein, and urea); high sensitive troponin T (hs-Troponin T); C-reactive protein (CRP); and blood gas results (base excess [BE], bicarbonate [HCO_3_], lactate, partial pressure of carbon dioxide, and pH). Blood samples are taken while establishing vascular access during CPR in out-of-hospital arrest cases and occur at the first opportunity during in-hospital arrest cases.

Since the duration of CPR performed by bystanders was not available during the screening, prehospital CPR was recorded only as the duration of CPR performed by emergency response teams.

### 2.4. Statistical Analysis

Data analysis was performed using IBM SPSS Statistics version 25 (IBM Corp., Armonk, NY, USA). The normality of the dataset distribution was evaluated based on skewness and kurtosis coefficients. Values between −2 and +2 were considered indicative of a normal distribution. Categorical variables were summarized according to the frequency and percentage; continuous variables by the mean, standard deviation, median, and first (Q1) and third (Q3) quartile values. For comparing continuous variables, Student’s *t*-test was used for normally distributed data, and the Mann–Whitney U test for non-normally distributed data. Binary logistic regression analysis was applied to determine factors associated with ROSC. The multicollinearity between variables was assessed using the variance inflation factor and tolerance values. Tolerance values above 0.1 and variance inflation factor values below 10 indicated no multicollinearity. To determine the classification power of laboratory variables that may differentiate mortality and ROSC status, individual receiver operating characteristic (ROC) analyses were conducted for each variable. ROC analyses were performed using MedCalc software version 23.0.6 (Ostend, Belgium), and area under the curve (AUC), sensitivity, specificity, and optimal cut-off values (associated criterion) were calculated for each variable. According to the ROC analysis, an AUC > 0.9 was considered excellent, 0.8 < AUC ≤ 0.9 good, 0.7 < AUC ≤ 0.8 moderate, 0.6 < AUC ≤ 0.7 poor, 0.5 < AUC ≤ 0.6 very poor, and AUC = 0.5 was a random classification. The threshold for statistical significance was set at *p* < 0.05, and the results were reported with odds ratios (ORs) and 95% confidence interval (CIs).

### 2.5. Ethics Committee Approval

Approval for this study was obtained from the ethics committee at our hospital (E-46059653-050.04-259056601, date: 8 November 2024).

## 3. Results

Among the 686 cases included in the study, spontaneous circulation (ROSC) was achieved in 13.6% (*n* = 93). The mean age of the patients was 67.7 ± 16.5 years, and 61.4% were male. When patients who experienced ROSC were compared to those with mortality in terms of chronic diseases, no significant difference was observed between the groups in relation to hypertension, diabetes mellitus, chronic renal failure, or coronary artery disease (*p* > 0.05). However, mortality was significantly higher among those with atrial fibrillation and neurodegenerative diseases compared to the ROSC group (*p* = 0.033 and *p* = 0.005, respectively).

When evaluated in terms of event characteristics, patients with shockable rhythms were significantly more likely to experience ROSC (*p* < 0.001). Regarding CPR duration, the in-hospital (*p* < 0.001) and total CPR duration (*p* < 0.001) were significantly shorter in the ROSC group compared to those without ROSC, whereas the prehospital CPR duration was significantly longer in the ROSC group (*p* = 0.003) (Table 1).

In regard to the comparison of the metabolic parameters among the patients, levels of HCO_3_, CRP, and creatinine were significantly lower in the ROSC group (*p* < 0.001, *p* < 0.001, and *p* = 0.025, respectively). In contrast, the albumin (*p* = 0.002) and glucose (*p* < 0.001) levels were significantly higher in the ROSC group. Regarding blood gas parameters, BE was significantly higher in the ROSC group (*p* < 0.001). In terms of hematologic parameters, levels of WBCs, neutrophils, and hemoglobin were significantly higher in the ROSC group (*p* < 0.001, *p* = 0.002, and *p* < 0.001, respectively), while the levels of lymphocytes, eosinophils, and RBCs were significantly lower (*p* = 0.006, *p* < 0.001, and *p* = 0.004, respectively) (Table 2).

According to the results of the binary logistic regression analysis conducted to identify predictors of ROSC in patients with cardiac arrest, the likelihood of ROSC was found to be 1.93 times higher in patients with a shockable cardiac rhythm compared to those with a non-shockable rhythm (*p* = 0.017, OR = 1.927, 95% CI: 1.126–3.297). Regarding metabolic parameters, each 1 mg/dL increase in the glucose level increased the likelihood of ROSC by 0.3% (*p* = 0.006, OR = 1.003, 95% CI: 1.001–1.004), while each 1 unit increase in BE raised the likelihood of ROSC by 21.4% (*p* < 0.001, OR = 1.214, 95% CI: 1.151–1.281). In contrast, for each 1 mmol/L increase in potassium (*p* = 0.042, OR = 0.798, 95% CI: 0.642–0.992) and HCO_3_ (*p* < 0.001, OR = 0.798, 95% CI: 0.745–0.855), the likelihood of ROSC decreased by 20.2%. Hematologically, each unit increase in the lymphocyte level was associated with an 11% decrease in the probability of ROSC (*p* = 0.029, OR = 0.890, 95% CI: 0.802–0.988), while each 1 g/dL increase in hemoglobin was associated with a 14.9% increase in ROSC probability (*p* = 0.001, OR = 1.149, 95% CI: 1.058–1.248) (Table 3).

**Figure 1:**
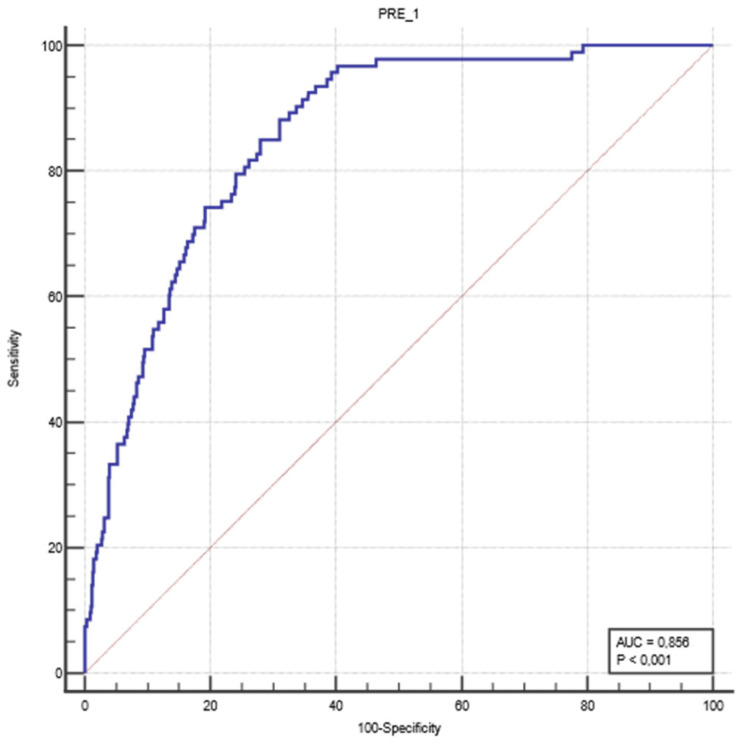
ROC curve to evaluate the classification performance of the logistic regression model. The blue line represents the ROC curve of the logistic regression model, and the red line indicates the reference line.

An ROC analysis was performed to evaluate the classification performance of the logistic regression model developed in the study. The predicted probability values generated for each case were compared with the actual ROSC outcomes. The analysis revealed that the model had an AUC value of 0.856, with a sensitivity of 88.2% and a specificity of 69.0% (Figure 1).

**Figure 2:**
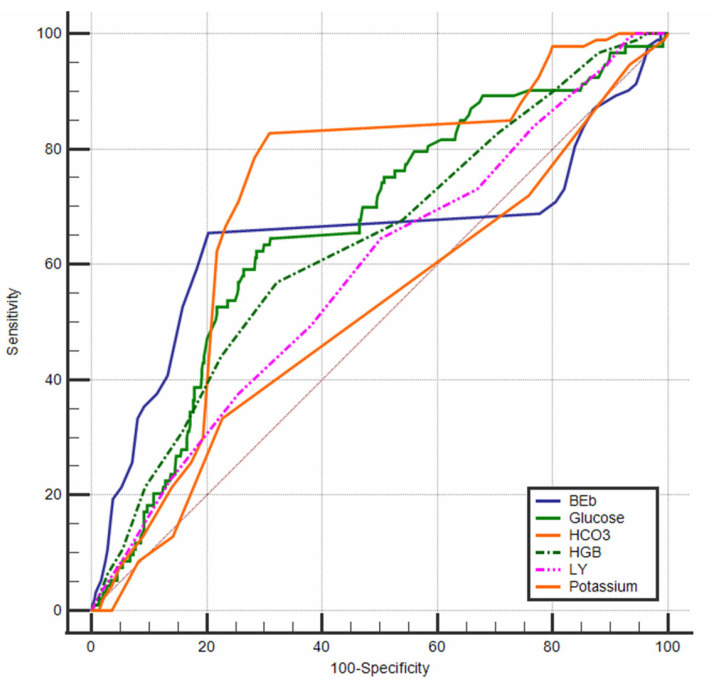
ROC curves of the laboratory findings.

An ROC analysis was also performed to determine the discriminative power of the variables included in the logistic regression model in distinguishing between mortality and ROSC (Figure 2). The analysis revealed that actual HCO_3_ values ≤ 19 mmol/L demonstrated a moderate level of discriminative power (AUC = 0.720, *p* < 0.0001). Similarly, when a threshold value of −18 was used, the variable BE (AUC = 0.647, *p* = 0.0002), as well as glucose levels > 205 mg/dL (AUC = 0.665, *p* < 0.0001), also showed moderate discriminative ability in identifying the ROSC status.

The study also revealed that hemoglobin levels above 11 g/dL demonstrated weak discriminative power (AUC = 0.636, *p* < 0.0001). With regard to lymphocyte ratios, values of 5% and below were found to offer limited discriminative ability (AUC = 0.584, *p* = 0.008). The findings indicated that potassium levels were not a statistically significant discriminator in the ROC analysis (*p* = 0.570) (Table 4).

## 4. Discussion

In this study, patients with a known diagnosis of atrial fibrillation were observed to have a higher mortality rate, while the rate of ROSC was higher among patients with a shockable rhythm and a shorter total CPR duration. Furthermore, the levels of albumin, CK, glucose, WBCs, neutrophils, monocytes, and hemoglobin were significantly higher in patients with successful ROSC, whereas the levels of creatinine, lymphocytes, eosinophils, RBCs, and BE were elevated in patients who had a fatal outcome.

In our study, we found that mortality was high in patients diagnosed with atrial fibrillation. Atrial fibrillation can cause stroke, heart failure, and various thromboembolic events [3]. Patients without appropriate anticoagulant use may be more prone to these events. Thromboembolic events increase both mortality and morbidity. Therefore, the higher mortality rate in patients with atrial fibrillation in our study may be due to adverse events related to AF.

Early achievement of spontaneous circulation and shorter resuscitation durations are associated with a favorable prognosis [4]. In our study, the prehospital CPR duration was longer in the ROSC group compared to those who did not achieve ROSC; however, the total CPR duration was shorter in the ROSC group. This observation may be attributed to effective CPR resulting in a quicker response, correction of the underlying etiology of cardiac arrest during CPR, improved perfusion in critically ill patients, and the absence of irreversible damage at the time of the intervention.

One of the essential components of high-quality CPR is early defibrillation. Early defibrillation during cardiac arrest is associated with higher ROSC and survival rates [5]. In a study by Park et al. on out-of-hospital cardiac arrests, it was found that cases in which ROSC was achieved in the field were younger, had received bystander CPR, and presented with a shockable rhythm. Age, initial rhythm, witnessed arrest, the location of the arrest, and response time were among the significant variables [6]. Similarly, a study conducted in the Netherlands by de Graaf et al., which included out-of-hospital cardiac arrest cases who received CPR by emergency medical services, showed that patients with ROSC were younger, more frequently presented with an initial shockable rhythm, and had witnessed arrests with CPR initiated by bystanders. In addition, defibrillation via automated external defibrillators (AEDs) was more commonly administered in ROSC cases. It was determined that cases defibrillated using an AED achieved ROSC in a shorter time compared to those defibrillated by emergency medical services or those who did not receive defibrillation. The time to ROSC was also shorter in non-fatal cases compared to those with mortality. The study found that achieving ROSC in a prehospital setting significantly improved survival outcomes in out-of-hospital cardiac arrest cases [7].

In a study by Okubo et al., including 348,996 patients with in-hospital cardiac arrest, ROSC was achieved in 66.9% of cases, and 22.6% were discharged from the hospital. It was observed that as the CPR duration increased, survival rates and favorable neurological outcomes decreased. Patients under 60 years of age, those with witnessed arrests, and those with an initial shockable rhythm achieved ROSC in a shorter time. It was concluded that younger, witnessed, and initially shockable rhythm patients benefited from longer durations of cardiopulmonary resuscitation compared to older, unwitnessed, and non-shockable rhythm cases [8]. In our study, similar mortality was observed in patients with longer CPR times and a higher incidence of non-shockable rhythms. However, due to the retrospective nature of our study, we could not obtain information on the neurological survival of the patients. This is a significant limitation of our study, as the primary goal of emergency services is to provide ROSC in cardiac arrest cases.

In-hospital cardiac arrest cases are associated with lower ROSC rates, lower discharge rates, and poorer neurological outcomes. In a study by Li et al., which included 851 patients with in-hospital cardiac arrest, the male sex, an age over 80 years, an arrest duration > 23 min, and a total adrenaline dose of >3 mg were identified as risk factors for a failure to achieve ROSC. Pre-arrest dysrhythmia, an initial shockable rhythm, and advanced airway placement were associated with ROSC. The CPR duration was longer in patients who failed to achieve ROSC or had poor neurological outcomes compared to other patient groups [2]. In our study, ROSC was found to be more common in out-of-hospital cardiac arrest cases. In in-hospital cardiac arrests, the likelihood of ROSC can be influenced by the patient’s knowledge of potential cardiac arrest pathologies, their ongoing monitoring and treatment, and the availability of all types of medical resources when an arrest is detected. Cases in which cardiac arrest develops despite the patient receiving treatment during hospitalization reduce the likelihood of ROSC, indicating both the severity of the underlying disease and the patient’s lack of response to treatment. Furthermore, our study found that the prehospital CPR duration was longer in the ROSC group. However, considering that the total CPR duration was shorter in the ROSC group, it suggests that the in-hospital CPR duration was shorter and resulted in a faster response. Therefore, the longer prehospital CPR duration in the ROSC group is not related to cardiac arrest itself, but rather to the time between the scene of the incident and the time the emergency team took to transport the patient to the hospital.

Chan et al. found that patients with ROSC were younger, of Caucasian ethnicity, had an initial shockable rhythm, and had fewer comorbidities and interventions during arrest [9]. In another study, Imamura et al. examined prognostic factors in cardiopulmonary arrest and reported that 30-day mortality was associated with the male sex, witnessed arrest, bystander CPR, cardiopulmonary arrest due to acute myocardial infarction, pupil size, the Glasgow Coma Scale score, the presence of pupillary light reflex, pH, lactate, the initial rhythm of ventricular fibrillation, and the duration of the arrest [10]. Another study reported that intubation and the use of mechanical compression devices during cardiac arrest were independent predictors of ROSC [11]. Consistent with these studies, our findings showed a higher incidence of ROSC in patients with a shockable rhythm and a lower probability of ROSC with prolonged CPR duration. In contrast to some previous studies, sex and age were not found to have a significant effect on mortality or ROSC. Moreover, unlike Imamura et al., we did not observe any association between pH or lactate levels and ROSC.

Upon evaluating the laboratory data of patients who achieved ROSC, we observed that lower glucose levels were associated with a more fatal course, which may relate to hypoglycemia, which is a metabolic cause of cardiac arrest, and prolonged CPR duration. The higher creatinine levels observed in the mortality group may be attributed to both renal hypoxia and the inability to meet other metabolic demands. Additionally, although the presence of chronic kidney disease does not appear to significantly affect ROSC or mortality rates, elevated creatinine levels may also reflect underlying chronic comorbidities. In our study, HCO_3_ levels were found to be lower in the ROSC group compared to the non-ROSC group, which may be attributed to the longer prehospital CPR duration in our ROSC group. In a study by Avci et al. evaluating predictors of ROSC, it was found that the platelet count was higher, the urea level was lower, the CPR duration was shorter, and the patients were younger in the ROSC group [12]. While our study did not reveal any significant differences in the platelet count, urea levels, or age between the ROSC and mortality groups, it was consistent in finding an association between a shorter CPR duration and ROSC. Moreover, the pH levels did not differ significantly between the groups, aligning with the findings in the previous study.

Laboratory parameters can provide information about the causes of cardiac arrest and can be used to evaluate the patient’s response to treatment after treating the underlying cause. At this stage, laboratory results can inform the physician in deciding whether to continue or terminate CPR. For patients presenting to the emergency department, data such as age, chronic diseases, acute illnesses, arrest-related data (arrest duration, shockable/non-shockable rhythms, IHCA/OHCA, etc.), and laboratory results can be recorded in hospital information management systems, and algorithms could be explored, using artificial intelligence to calculate the likelihood of ROSC in future studies. While the final decision remains with the physician, this approach could support physicians during decision making. Furthermore, although the laboratory tests we used in our study are readily available, they may not always yield results quickly due to limited resources at healthcare centers or various disruptions that occur during the process. This may limit the practical application of the data we obtained in our study.

In summary, considering the quality of CPR, earlier chest compressions and faster defibrillation correlate with higher ROSC rates. Therefore, in cases of out-of-hospital cardiac arrest, public education in regard to basic life support is of critical importance. Furthermore, given the increased likelihood of ROSC in patients with shockable rhythms and the benefits of early defibrillation, it is essential to promote the widespread availability and use of AEDs in prehospital settings, as well as provide targeted training on their use. In addition to expanding AED access outside hospitals, equipping AED stations with tools such as bag–valve masks and supraglottic airway devices may improve the continuity of ventilation during CPR. As such, incorporating training on oral airways, bag–valve masks, and supraglottic airways into basic life support courses for lay rescuers may be beneficial. Ensuring advanced airway placement by emergency medical services can help address reversible hypoxia, a known cause of cardiac arrest, and increase ROSC rates. While mechanical CPR devices are not yet routinely recommended in current basic and advanced life support guidelines, they may improve the effectiveness and consistency of chest compressions during ambulance transport, particularly in scenarios involving high patient volume, limited personnel, or simultaneous resuscitation of multiple patients. Further research is needed to evaluate the routine use of mechanical compression devices in emergency departments, as they are not yet used as part of standard practice.

## 5. Conclusions

This study identified laboratory variables, such as creatine kinase, HCO_3_, CRP, glucose, creatinine, WBCs, neutrophils, monocytes, lymphocytes, eosinophils, RBCs, hemoglobin, and BE as potentially informative markers for predicting ROSC or mortality. The integration of these variables into clinical algorithms may support decision making in crowded emergency departments.

Accurate ROSC prediction is crucial in overcrowded emergency departments for the efficient allocation of personnel and other resources. Therefore, there is a need for further studies and models aimed at predicting ROSC.

Our study has certain limitations. The retrospective and single-center design constitutes the primary limitation. Additionally, due to the retrospective nature of the study, data on neurological survival and long-term outcomes of the patients who achieved ROSC could not be obtained. In our study, the prevalence of bystander CPR and, if performed, the duration of the CPR were not available, which represents another limitation of our study.

## Figures and Tables

**Table 1:** Demographic, clinical, and event characteristics of the sample.

	Mortality (*n* = 593)	ROSC (*n* = 93)	Total	*p*
Demographic and clinical characteristics				
Age, mean ± SD	67.8 ± 16.6	67.1 ± 16.0	67.7 ± 16.5	0.713
Male, *n* (%)	364 (86.5)	57 (13.5)	421 (61.4)	0.986
DM, *n* (%)	117 (84.2)	22 (15.8)	139 (20.3)	0.381
HT, *n* (%)	177 (83.9)	34 (16.1)	211 (30.8)	0.192
CAD, *n* (%)	77 (81.1)	18 (18.9)	95 (13.8)	0.098
CHF, *n* (%)	64 (86.5)	10 (13.5)	74 (10.8)	0.991
CRF, *n* (%)	27 (84.4)	5 (15.6)	32 (4.7)	0.790
Cancer, *n* (%)	43 (81.1)	10 (18.9)	53 (7.7)	0.240
COPD/asthma, *n* (%)	58 (86.6)	9 (13.4)	67 (9.8)	0.975
CVA, *n* (%)	20 (74.1)	7 (25.9)	27 (3.9)	0.078
AF/dysrhythmia, *n* (%)	13 (68.4)	6 (31.6)	19 (2.8)	0.033
Aortic valve pathology, *n* (%)	2 (0.5)	2 (0.5)	4 (0.6)	0.091
Event characteristics				
IHCA, *n* (%)	251 (92.6)	20 (7.4)	271 (40)	<0.001
OHCA, *n* (%)	332 (82)	73 (18)	405 (60)	
Shockable cardiac rhythm, *n* (%)	124 (77.0)	37 (23.0)	161 (23.5)	<0.001
Non-shockable cardiac rhythm, *n* (%)	469 (89.3)	56 (10.7)	525 (76.5)	
CPR duration (min)				
In-hospital, median (Q1–Q3)	30 (30.0–35.5)	15 (15.0–24.5)	30 (25.0–35.0)	<0.001
In-hospital (IHCA), median (Q1–Q3)	30 (30–35)	16 (9.5–30)		<0.001
In-hospital (OHCA), median (Q1–Q3)	34 (30–40)	15 (5–24)		<0.001
Prehospital, mean ± SD	9.5 ± 11.1	13.2 ± 10.3	10.0 ± 11.1	0.003
Prehospital (OHCA), median (Q1–Q3)	15 (10–20)	15 (13–20)		0.434
Total CPR duration, mean ± SD	40.0 (30.0–50.0)	30.0 (17.0–40.0)	40.0 (30.0–50.0)	<0.001
Total CPR (IHCA), median (Q1–Q3)	30 (30–35)	16 (9.5–30)		<0.001
Total CPR (OHCA), median (Q1–Q3)	50 (40–75)	30 (20.5–41)		<0.001

CAD: coronary artery disease, CHF: congestive heart failure, COPD: chronic obstructive pulmonary disease, CRF: chronic renal failure, AF: atrial fibrillation, CVA: cerebrovascular accident, IHCA: in-hospital cardiac arrest, OHCA: out-of-hospital cardiac arrest.

**Table 2:** Laboratory findings.

	Mortality (*n* = 593)	ROSC (*n* = 93)	Total	*p*
pH	7.0 ± 0.2	7.0 ± 0.2	7 ± 0.2	0.421
HCO_3_act (mmol/L)	19.8 ± 6.7	15.6 ± 4.5	19.3 ± 6.6	<0.001
pCO_2_ (mmHg)	62.2 (43.5–68.5)	60.0 (42.0–81.7)	62.0 (43.2–69.9)	0.573
Lactate (mmol/L)	11.6 ± 4.3	10.6 ± 5.5	11.5 ± 4.5	0.85
BE (mmol/L)	−18 (−18 to −18)	−15.2 (−20.8 to −9.9)	−18 (−18 to −16.8)	<0.001
Albumin (g/L)	31.4 ± 6.9	33.8 ± 7.4	31.7 ± 7.1	0.002
Protein (g/L)	6.1 ± 0.9	6.1 ± 0.9	6.1 ± 0.9	0.807
CRP (mg/L)	49 (13.6–79.6)	18.5 (3.3–68.9)	45.4 (11.3–79.1)	<0.001
ALT (U/L)	37 (25–72.5)	39 (21.1–106.0)	38 (25–75)	0.888
AST (U/L)	63 (35–146)	52 (28–99.2)	61 (34–146)	0.317
CK (U/L)	123 (70.5–200)	141 (84.5–268)	128 (73–200)	0.019
Glucose (mg/dL)	156 (96–239)	256 (146.0–319.0)	173 (98–256)	<0.001
Calcium (mmol/L)	8.6 (8.4–9.4)	9.0 (8.4–9.5)	8.7 (8.4–9.4)	0.108
Chlorine (mmol/L)	100 (96.7–104.0)	99.3 (95.4–103.4)	100 (96.7–103.7)	0.452
Creatinine (mg/dL)	1.5 (1.0–1.9)	1.2 (1.0–1.6)	1.4 (1.0–1.9)	0.025
Potassium (mmol/L)	5.4 (4.6–5.4)	5.2 (4.4–5.9)	5.4 (4.5–5.4)	0.230
Sodium (mmol/L)	138 (135–142)	139 (135–145)	138 (135–142)	0.197
Urea (mg/dL)	58 (35–60)	58 (33–63)	58 (34–60)	0.445
Troponin (ng/mL)	59.8 (29–146)	61 (26.7–98.5)	60 (28.9–146)	0.310
WBC (10^3^/µL)	11 (7.9–13.8)	12.5 (9.9–16.1)	11.3 (8.2–14.1)	<0.001
Neutrophils (10^3^/µL)	5.5 (3.6–7.3)	7.1 (3.9–9.7)	5.5 (3.7–7.7)	0.002
Monocytes (10^3^/µL)	0.5 (0.3–0.7)	0.6 (0.4–0.9)	0.5 (0.3–0.7)	0.001
Lymphocytes (10^3^/µL)	5.5 (3.5–7.3)	4.5 (2.6–6.8)	5.3 (3.3–7.2)	0.006
Eosinophils (10^3^/µL)	0.2 (0.1–0.4)	0.1 (0–0.2)	0.2 (0.1–0.3)	<0.001
RBC (10^6^/µL)	4.6 (3.7–5.4)	4.2 (3.5–4.9)	4.5 (3.7–5.3)	0.004
Hemoglobin (g/dL)	10.6 (9.0–12.1)	12.1 (10.1–14.2)	10.6 (9.1–12.5)	<0.001
Hematocrit (%)	37.2 ± 8.4	38.3 ± 8.0	37.4 ± 8.4	0.235
Platelet count (10^3^/µL)	204 (154.5–249.5)	207 (152–269.5)	204 (154–252.3)	0.278
BE (mmol/L)	−18 (−18 to −18)	−15.2 (−20.8 to −9.9)	−18 (−18 to −16.8)	<0.001

HCO_3_act: actual bicarbonate, pCO_2_: partial pressure of carbon dioxide, CRP: C-reactive protein, ALT: alanine aminotransferase, AST: aspartate aminotransferase, CK: creatine kinase, WBC: white blood cell, RBC: red blood cell, BE: base excess.

**Table 3:** Predictors of a return of spontaneous circulation in patients with cardiac arrest.

	B	Wald	*p*	Exp(B)	95% CI	
					Lower	Lower
Age	−0.002	0.066	0.797	0.998	0.982	1.014
Prehospital CPR duration	0.022	3.562	0.059	1.022	0.999	1.046
Creatine kinase	0.000	1.613	0.204	1.000	1.000	1.001
Glucose	0.003	7.464	0.006	1.003	1.001	1.004
Potassium	−0.225	4.129	0.042	0.798	0.642	0.992
HCO_3_act	−0.226	41.673	<0.001	0.798	0.745	0.855
BE	0.194	49.989	<0.001	1.214	1.151	1.281
Lymphocytes	−0.117	4.760	0.029	0.890	0.802	0.988
Hemoglobin	0.139	10.776	0.001	1.149	1.058	1.248
Cardiac rhythm						
Shockable cardiac rhythm	0.656	5.733	0.017	1.927	1.126	3.297
Non-shockable cardiac rhythm	Reference					
Constant	4.563	11.656	0.001	95.871		

CI: confidence interval, HCO_3_act: actual bicarbonate, BE: base excess, CPR: cardiopulmonary resuscitation. Cox and Snell R^2^ = 0.192, Nagelkerke R^2^ = 0.350, accuracy = 0.87, χ^2^ = 146.039, *p* < 0.001.

**Table 4:** ROC analysis of the variables included in the regression model.

Variables	AUC	*p*	Youden İndex J	Associated Criterion	Sensitivity	Specificity
BE (mmol/L)	0.647	0.0002	0.4519	>−18	65.59	79.60
Glucose (mg/dL)	0.665	<0.0001	0.3370	>205	62.37	71.33
HCO_3_act (mmol/L)	0.720	<0.0001	0.5177	≤19	82.80	68.97
Hemoglobin (g/dL)	0.636	<0.0001	0.2478	>11	56.99	67.79
Lymphocytes (×10^9^/L)	0.584	0.008	0.1426	≤5	64.52	49.75
Potassium (mEq/L)	0.518	0.570	0.1057	>5	33.33	77.23

BE: base excess, HCO_3_act: actual bicarbonate.

## Data Availability

Dataset available on request from the authors. The datasets are not publicly available due to privacy.

## Abbreviations

The following abbreviations are used in this manuscript:

CPR	Cardiopulmonary resuscitation
ROSC	Return of spontaneous circulation
ROC	Receiver operating characteristic
WBC	White blood cell
RBC	Red blood cell
CK	Creatine kinase
CRP	C-reactive protein
BE	Base excess
HCO_3_	Bicarbonate
AUC	Area under the curve
OR	Odds ratio
CI	Confidence interval
IHCA	In-hospital cardiac arrest
OHCA	Out-of-hospital cardiac arrest
